# Neither non-toxigenic *Staphylococcus aureus* nor commensal *S. epidermidi* activates NLRP3 inflammasomes in human conjunctival goblet cells

**DOI:** 10.1136/bmjophth-2017-000101

**Published:** 2017-11-16

**Authors:** Dayu Li, Robin R Hodges, Paulo Bispo, Michael S Gilmore, Meredith Gregory-Ksander, Darlene A Dartt

**Affiliations:** 1 Schepens Eye Research Institute/Massachusetts Eye and Ear, Boston, Massachusetts, USA; 2 Department of Ophthalmology, Harvard Medical School, Boston, Massachusetts, USA; 3 Massachusetts Eye and Ear, Boston, Massachusetts, USA; 4 Department of Microbiology and Immunobiology, Harvard Medical School, Boston, Massachusetts, USA

**Keywords:** inflammation, infection

## Abstract

**Purpose:**

The conjunctiva is a wet mucosal surface surrounding the cornea that is continuously exposed to pathogens. Nevertheless, persistent inflammation is not observed. We examined if the NOD-like receptor pyrin domain 3 (NLRP3) inflammasome functions as a sensor that distinguishes commensal and non-pathogenic bacteria from pathogenic bacteria in human conjunctival goblet cells.

**Methods:**

Goblet cells were grown from human conjunctiva and co-cultured with commensal *Staphylococcus epidermidis*, isogenic non-toxigenic *S. aureus* ACL135 and as a control toxigenic *S. aureus* RN6390. Activation of the NLRP3 inflammasome was determined by measuring changes in NF-κB activity, expression of pro-interleukin (IL)-1β and NLRP3, activation of caspase-1 and secretion of mature IL-1β. Goblet cell mucin secretion was measured in parallel.

**Results:**

While all three strains of bacteria were able to bind to goblet cells, neither commensal *S. epidermidis* nor isogenic non-toxigenic *S. aureus* ACL135 was able to stimulate an increase in (1) NF-κB activity, (2) pro-IL-1β and NLRP3 expression, (3) caspase-1 activation, (4) mature IL-1β and (5) mucin secretion. Toxigenic *S. aureus,* the positive control, increased these values: knockdown of NLRP3 with small interfering RNA (siRNA) completely abolished the toxigenic *S. aureus*-induced expression of pro-IL-1β and secretion of mature IL-1β.

**Conclusions:**

We conclude that NLRP3 serves as a sensor capable of discriminating commensal and non-pathogenic bacteria from pathogenic bacteria in conjunctival goblet cells, and that activation of the NLRP3 inflammasome induced by pathogenic bacteria mediates secretion of both mature IL-1β and large secretory mucins from these cells.

**Key messages**

The conjunctiva is normally colonised with commensal bacteria, and constitutively expresses the NOD-like receptor pyrin domain 3 (NLRP3) inflammasome. In spite of these, the conjunctiva is not normally inflamed. In this study, we found that NLRP3 serves as a sensor capable of discriminating commensal and non-pathogenic bacteria from pathogenic bacteria in conjunctival goblet cells, and that activation of the NLRP3 inflammasome induced by pathogenic bacteria mediates secretion of both mature interleukin-1β and large secretory mucins from these cells. In situations in which NLRP3 continues to be active in the absence of bacteria, inhibition of NLRP3 could be used to treat this inflammation.

## Introduction

The mucosal epithelium acts as both a physical and an immunological barrier, limiting access of pathogenic bacteria and their products to the underlying epithelium. The host response to pathogens relies on detection of pathogens by pattern recognition receptors, such as Toll-like receptors (TLRs) and Nod-Like receptors (NLRs).^1^ These receptors respond to microbial pathogen-associated molecular patterns (PAMPS), and to altered host molecules known as danger-associated molecular patterns (DAMPS). Activation of these receptors results in production of multiple proinflammatory mediators that coordinate a response ensuring removal or death of the pathogen.^2^ However, wet surface mucosal epithelia, such as the conjunctiva, are continuously exposed to both commensals and pathogens, and yet limit the inflammatory response.

Compared with the gut and respiratory tract, the ocular surface is associated with a more limited microbiota, with <100 CFU/µL typically shed in tears, compared with 10^7^–10^8^ CFU/µL in saliva.^3^ This small population of bacteria does not usually cause ocular surface disease. In tears, commensal *Staphylococcus epidermidis* usually predominate, but *S. aureus* is also frequently present.^3^ *S. aureus* is the leading cause of ocular surface infection, independent of contact lens wear.^4 5^ Recent studies in the gut demonstrate that interaction between TLRs and commensal bacteria is critical for maintaining the mucosal barrier to infection.^6^ Cytoplasmic receptors, such as NLRs, recognise intracellular PAMPs and DAMPs released following damage to host tissue. Thus, activation of NLRs contributes to activating innate pathways of inflammation.^7^

Goblet cells serve as an important component of the innate immune system, and recent studies show that they have much in common with other immune cells.^8 9^ Inflammasomes are critical mediators of innate immunity, and are constitutively expressed in epithelial goblet cells. The NOD-like receptor pyrin domain 3 (NLRP3) inflammasome is expressed by conjunctival goblet cells,^9^ whereas the NLRP6 but not the NLRP3 inflammasome is expressed by intestinal goblet cells.^10^ Goblet cells of the small intestine and more recently the conjunctiva have been shown to be important for intestinal immune homeostasis by delivering antigens from the intestinal lumen and ocular surface, respectively, to the underlying dendritic cells.^10 11^

The large gel-forming mucin MUC5AC is secreted by conjunctival goblet cells, but not the other epithelial cell type in the conjunctiva, the stratified squamous cells.^12^ This mucin provides a physical barrier and possesses antibacterial properties. MUC5AC expression is stimulated by inflammation.^13 14^ Ocular surface inflammation is triggered in part through stimulation of TLRs.^15 16^ All TLRs except TLR8 are present in the conjunctiva.^17^ TLR2, TLR3, TLR4, TLR5 and TLR9 were found to be activated by bacterial products in either the conjunctiva or cornea.^16 17^ We previously described constitutive expression of TLRs 1, 2 and 6 by conjunctival goblet cells.^9^

TLR signalling is the first step in the innate immune response to pathogens, triggering transcriptional induction of pro-interleukin (IL)-1β and pro-IL-18. However, NLR signalling is required for the proteolytic processing of pro-IL-1β and pro-IL-18 through formation of multiprotein complexes, known as inflammasomes.^18^ The best characterised NLR gene is NLRP3, and the NLRP3 inflammasome is a signalling complex that activates procaspase-1 and induces the proteolytic processing of IL-1β and IL-18. We previously demonstrated that NLRP3 is constitutively expressed by human and rat conjunctival goblet cells.^9^ Moreover, toxigenic *S. aureus* activates the NLRP3 inflammasome in rat conjunctival goblet cells to produce mature, active IL-1β.^9^

In spite of the colonisation of the conjunctiva with commensal bacteria, and the constitutive expression of the NLRP3 inflammasome,^3 9^ the conjunctiva is not normally inflamed. To understand how the mucosal barrier is differentially affected by commensal, non-pathogenic and pathogenic bacteria, and to determine how inflammation is induced, we asked whether the NLRP3 inflammasome of human conjunctival goblet cells serves as a sensor capable of discriminating non-pathogenic from pathogenic bacteria.

## Materials and methods

### Human tissue

Donor conjunctival tissue was obtained from Heartland Lions Eye Bank (Kansas City, Missouri, USA) and Eversight (Ann Arbor, Michigan, USA) for research purposes. No consent was obtained as no identifying information was transferred. Tissue was placed in Optisol medium within 18 hours of death. Conjunctiva was cleaned of connective tissue before use. Conjunctiva from three different individuals was used for each type of experiment.

### Cell culture

Goblet cells were grown in organ culture from human conjunctiva, as described previously.^19^ Briefly, pieces of minced tissue were placed in RPMI 1640 medium supplemented with 10% fetal bovine serum (FBS), 2 mM glutamine (Lonza, Walkersville, Maryland, USA) and 100 mg/mL penicillin/streptomycin in six-well plates. After nodules of cells appeared, the tissue plug and non-goblet cells were removed. After 7 days, the goblet cells were trypsinised and plated in 6, 24 or 96-well plates. As previously published,^9 20–23^ cultured cells were identified as goblet cells by the following methods: (1) morphology by light microscopy in bright field that indicates numerous small clear granules and positive staining with periodic acid-Schiff’s reagent that stains the secretory product blue and magenta; (2) positive staining with the lectin Helix pomatia agglutinin (HPA) and with an antibody to the large-gel forming mucin MUC5AC that both identify the secretory product; and (3) positive staining with the cytokeratin (CK) 7 that detects keratin filaments in goblet cell bodies and negative staining with CK 4 that detects keratin in stratified squamous cells.

### Cell viability measurements

To determine the number of live cells, first-passage human conjunctival goblet cells were seeded in 48-well plates. After incubation with bacteria for 1–6 hours, cultured cells were removed by tryspsin digestion. Trypan blue (Sigma-Aldrich, St. Louis, Missouri, USA) was added to trypsinised cells to a final concentration of 0.5% (w/v). The number of live cells was determined using a hemocytometer.

### Adhesion assay

Primary human goblet cells were grown to approximately 90% confluence in 24-well plates at 37°C in 5% CO_2_. Cell monolayers were washed three times with RPMI containing no antibiotics or FBS. Fresh overnight bacterial cultures on blood agar were grown in tryptic soy broth to an OD_600_ of 0.5 and washed twice with phosphate buffered saline (PBS). Pellets were resuspended in RPMI without antibiotics and FBS, and then added to the goblet cells at 10^6^ CFU/mL. Cells were incubated for 1 hour with bacteria at 37°C in 5% CO_2_ and then washed three times with PBS to remove non-attached bacteria. Dissociation of the cells was achieved with trypsin, and the resulting solution was serially diluted in PBS. Each dilution was plated on blood agar and incubated overnight at 37°C for colony counting. Adhesion was performed in triplicate and repeated three times. The methicillin-resistant *Staphylococcus aureus* (MRSA) strain USA300, known to bind to epithelial cells, was used as a positive control.^24 25^ Results are percentage of adherent bacteria on goblet cells.

### Bacterial culture and challenge of human conjunctival goblet cells

Three different bacteria were used in this study. The *S. epidermidis* strain 1947 was used as a representative of commensal flora. *S. epidermidis* was obtained from G. Pier (Harvard Medical School, Boston, Massachusetts, USA). *S. aureus* strain ACL135, which was derived from *S. aureus* RN6390, is an isogenic attenuated strain defective in the key global regulators *agr* and *sar*,^26 27^ and as a result produces few *S. aureus* toxins. *S. aureus* strain ACL135 was used to control for the inflammogenic activity of all other cellular constituents (eg, peptidoglycan, teichoic acids, and so on), aside from secreted toxins in *S. aureus* RN6390. The toxigenic strain *S. aureus* RN6390^28^ was used as a positive control. All bacteria were grown at 37°C to an OD595 nm of 0.5 (early log phase). After centrifugation at 500 × g for 10 min, the supernatant was discarded and bacteria were suspended in fresh RPMI-1640 medium with 1% FBS.

First-passage human conjunctival goblet cells were seeded in culture plates in medium without antibiotics, 24 hours prior to infection. The three strains of bacteria were added at a multiplicity of infection (MOI) of 20 or 60, and incubated for 1 to 6 hours at 37°C in 5% CO_2._ The incubation time and amount of bacteria was kept low in our experiments to minimise the effect of cell death and release of intracellular components. However, non-toxigenic bacteria activation of caspase-1 showed that this strain of bacteria was active at the conditions used.

### Measurement of NF-κB activity

Goblet cell nuclear extracts were isolated after a 4-hour infection with each of the bacteria. The cells were washed with PBS containing phosphatase inhibitor solution (Cayman Chemical Company, Ann Arbor, USA) and collected using a rubber policeman. The nuclear proteins were extracted according to the manufacturer’s instructions (Cayman Chemical Company). The nuclear fraction was assayed for NF-κB subunit activity, which was determined using an NF-κB (p65) transcription factor assay kit (Cayman Chemical Company).

### Western blotting analysis

First-passage human conjunctival goblet cells were seeded in six-well plates. After incubation with the three strains of bacteria, supernatant was removed and cells were lysed in RIPA buffer (10 mM Tris-HCl (pH 7.4), 150 mM NaCl, 1% deoxycholic acid, 1% Triton X100, 0.1% SDS and 1 mM EDTA). Protein was collected for analysis by western blot. Briefly, the lysate was centrifuged at 10 000 × *g* for 10 min at 4°C. Sample buffer was added to the lysate, proteins separated by SDS-PAGE through 10% polyacrylamide and transferred to nitrocellulose membrane to be processed for western blot. The primary antibodies were used for western blots at the following dilutions: 1:200 NLRP3, 1:400 pro-IL1β and 1:1000β-actin. NLRP3 and pro-IL-1β antibodies were purchased from R&D Systems (Minneapolis, Minnesota, USA), and β-actin antibody from Sigma Aldrich (St. Louis). Pro-IL-1β and NLRP3 were normalised to β-actin. For detection, secondary antibodies used were donkey anti-sheep (R&D Systems) and chicken anti-goat (EMD Millipore, Billerica, Massachusetts) conjugated to horseradish peroxidase. Immunoreactive bands were visualised by the Enhanced Chemiluminescence method (Thermo Scientific, Rockford, Illinois, USA). Results were expressed as fold increase above the normalised basal level.

### Knockdown of NLRP3

First-passage human goblet cells were cultured in RPMI 1640 with 10% FBS. A mixture of four predesigned NLRP3 siRNAs (Smart Pool) were obtained from Thermo Scientific Dharmacon RNAi Technologies (Lafayette, Colorado, USA). Transfection of siRNA was performed with the DharmaFECT 1 siRNA Transfection Reagent following the manufacturer’s protocol. siRNAs constructs were added at a final concentration of 0–100 nM for NLRP3 siRNA in antibiotic-free RPMI 1640 as described previously.^29^ Scrambled siRNA (sc-siRNA, 100 nM) was used as a negative control. The cells transfected with siRNA were cultured for 48 hours before experimental analysis.

### Mature IL-1β secretion

Supernatants from cells in which pro-IL-1β was measured were used to determine mature IL-1β by ELISA (R&D Systems), which was performed according to the manufacturer’s instructions. The ELISA detected mature IL-1β over a range of 1–2500 pg/mL. Values were expressed as fold increase over basal (no additions).

### FLICA assay for active caspase-1 analysis

Active caspase-1 was detected using a fluorescent inhibitor of caspases (FAM-FLICA Caspase 1 Assay Kit, Immunochemistry Technologies, Bloomington, Minnesota) according to the manufacturer’s instructions. First-passage human goblet cells were grown in 96-well plates. Before use media was changed to antibiotic-free RPMI media containing 1% FBS. The cells were then were stimulated with bacteria for 4 hours and 10 µL of 30X FLICA solution added. Following incubation, cells were stained with Hoechst 33 342 stain (0.5% w/v) and viewed on an inverted phase-contrast microscope equipped for epifluorescence (Eclipse TE300, Nikon, Tokyo, Japan) with a UV filter with excitation 490 nm, emission >520 nm for green fluorescence for caspase-1 positive cells and excitation 365 nm, emission 480 nm for visualisation of nuclei stained with the Hoechst dye. The total number of nuclei in four ×400 fields of view was counted, and the number of cells staining green (indicated active caspase-1) was expressed as a percentage of the total number of cells. For each experiment, an exposure time was set for the basal condition and held constant for the conditions in which bacteria were present.

### Measurement of high-molecular-weight glycoconjugate secretion

First-passage human conjunctival goblet cells were seeded on 24-well plates and grown to confluency. Cultured cells were serum-starved in RPMI-1640 supplemented with 0.5% BSA for 2 hours before use, and then stimulated with bacteria for 0.5–8 hours. Goblet cell secretion was measured using an enzyme-linked lectin assay with the lectin Ulex Europaeus Agglutinin (UEA-1). UEA-1 detects high-molecular-weight glycoconjugates, including mucins synthesised and secreted by human goblet cells. The media were collected and analysed for the amount of lectin-detectable glycoconjugates to quantify goblet cell secretion that includes mucins as previously published.^21–23 30–32^ The amount of glycoprotein secretion was standardised to the amount of total protein in each well as determined by the Bradford assay.^33^ Glycoconjugate secretion was expressed as fold increase.

## Statistical analysis

Results are presented as mean ±SEM. Data were analysed for significance using Student’s *t* test. A p value of <0.05 was considered statistically significant.

## Results

### Comparative viability of conjunctival goblet cells and bacterial cell adhesion with commensal non-toxigenic and toxigenic bacteria

**Figure 1:**
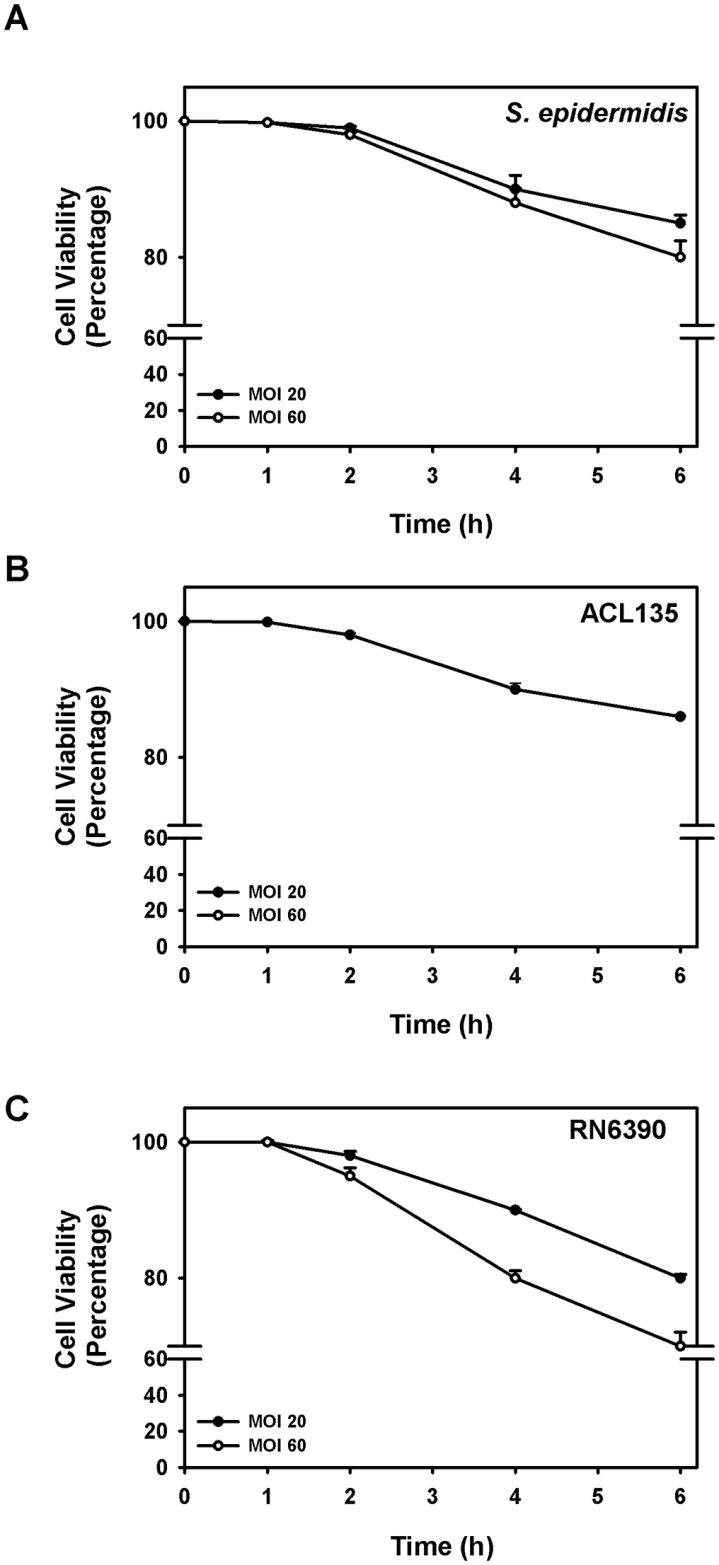
Goblet cell viability with *Staphylococcus epidermidis*, *S. aureus* ACL 135 and*S. aureus* RN6390. Cultured human goblet cells were incubated for 0–6 hours with *S. epidermidis* (A), *S. aureus* ACL135 (B) or *S. aureus* RN6390 (C) at an MOI of 20 (solid circles) and 60 (open circles), and cell viability was determined using the trypan blue exclusion assay. Data are expressed as mean±SEM from three independent experiments. No significant differences were detected. MOI, multiplicity of infection.

Co-culture of human conjunctival goblet cells with *S. epidermidis* (commensal), *S. aureus* ACL135 (non-toxigenic) and *S. aureus* RN6390 (toxigenic, the positive control) was first optimised to permit maximum stimulation of epithelial cells without inducing measurable cell death (figure 1). Human goblet cells were challenged with *S. epidermidis*, *S. aureus* ACL135 or *S. aureus* RN6390 at MOIs of either 20 or 60, and cell viability was assessed over 6 hours by trypan blue exclusion (figure 1).^9^ Seeding human goblet cell cultures with *S. epidermidis*, *S. aureus* ACL135 or *S. aureus* RN6390 at an initial MOI of 20 resulted in >90% goblet cell viability after a 4-hour incubation in all groups (figure 1), with between 80% and 90% of goblet cells surviving after 6 hours. While no significant differences in cell viability of goblet cells exposed to any of the three strains of bacteria were noted at 1, 2, 4 and 6 hours compared with 0 hour, the 4-hour endpoint with >90% goblet cell viability in all groups was chosen for subsequent experiments.

**Figure 2:**
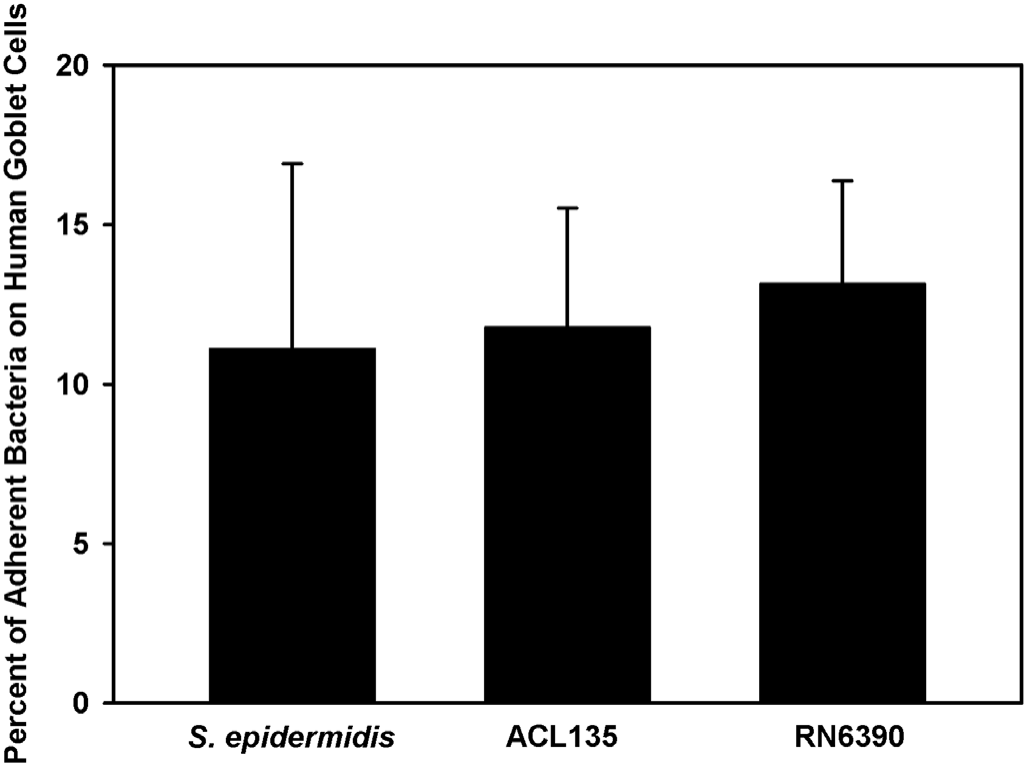
Bacterial adhesion to goblet cells with *Staphylococcus epidermidis*, *S. aureus* ACL 135 and *S. aureus* RN6390. Cultured human goblet cells were incubated with each strain of bacteria at a multiplicity of infection of 20 for 4 hours and rinsed to remove non-adhered bacteria. Cells were dissociated, plated on blood agar and incubated overnight. The resulting colonies were counted. Data are expressed as mean±SEM from three independent experiments.

Because bacteria differ in their ability to adhere to eukaryotic cells, bacterial adherence to goblet cells was measured with all three strains of bacteria at an MOI of 20 for 4 hours (figure 2). Adherence of both *S. aureus* ACL135 and *S. epidermidis* (11.8%±3.7% and 11.1%±5.8%, respectively) was not significantly different from that of *S. aureus* RN6390 that was 13.2%±3.2%.

### Effect of commensal, non-toxigenic and toxigenic bacteria on NF-κB activity and production of pro-IL-1β in conjunctival goblet cells

**Figure 3:**
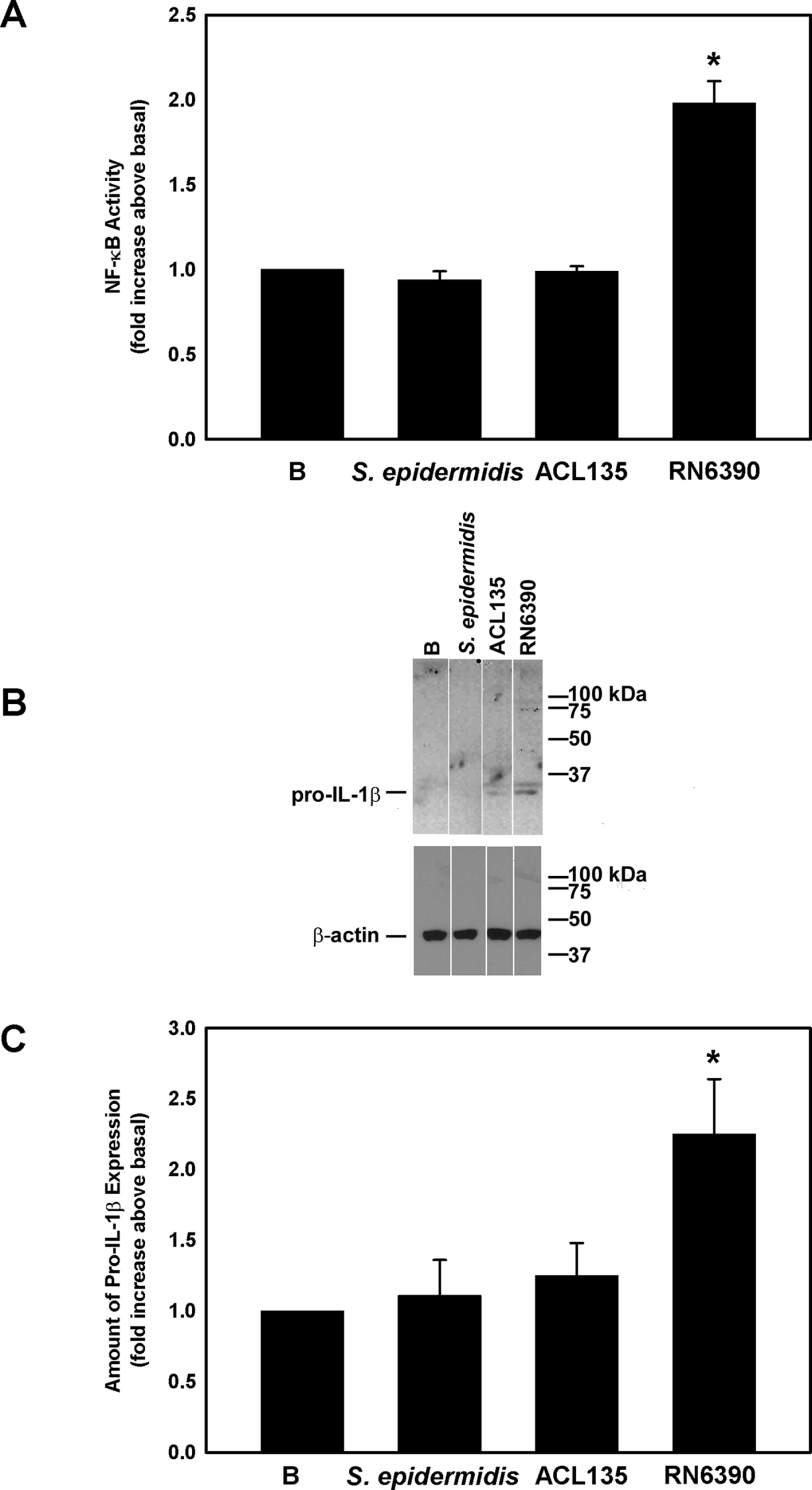
Effect of *Staphylococcus epidermidis*, *S. aureus* ACL 135 and *S. aureus* RN6390 on NF-κB activity and amount of pro-IL-1β. Cultured human goblet cells were cultured alone for 4 hours (basal (B)) or co-cultured for 4 hours with *S. epidermidis*, *S. aureus* ACL135 or *S. aureus* RN6390 at a multiplicity of infection of 20. The activity of NF-κB was measured in the cell lysates and is presented as fold increase above basal (B) in (A). The amount of pro-IL-1β was also measured by western blot. A representative blot is shown in (B). The amount of pro-IL-1β was standardised to the amount of β-actin and presented as fold increase above basal in (C). Data are expressed as mean±SEM from three independent experiments. * indicates significance from basal (B). IL, interleukin.

Several studies demonstrate that activation of the NLRP3 inflammasome and release of mature IL-1β requires two steps: (1) a priming step mediated by TLR2, resulting in NF-κB activation and production of pro-IL-1β and NLRP3, and (2) a second stimulus that triggers the assembly of the inflammasome and the activation of caspase-1.^34 35^ Activation of TLR-dependent signalling components was assessed in human conjunctival goblet cells co-cultured with *S. epidermidis*, *S. aureus* ACL135 or *S. aureus* RN6390 for 4 hours at an MOI of 20 (figure 3). Neither *S. epidermidis* nor *S. aureus* ACL135 induced NF-κB activity (1.0±0.03-fold and 0.9±0.05-fold increase over basal, respectively). In contrast, co-culture with the toxigenic *S. aureus* RN6390, the positive control, resulted in a significant increase in NF-κB activity by 2.0±0.1-fold over basal (figure 3A).

Western blot was used to assess expression of pro-IL-1β in cell lysates prepared from human conjunctival goblet cells cultured under the same conditions used to measure NF-κB activity. Pro-IL-1β was detected as a major band at 31 kDa (figure 3B). There was no significant increase in pro-IL-1β levels in conjunctival goblet cells co-cultured with *S. epidermidis* (1.1±0.3-fold increase over basal) or *S. aureus* ACL135 (1.3±0.2-fold over basal). By contrast, co-culture of human conjunctival goblet cells with the toxigenic *S. aureus* RN6390, the positive control, significantly increased the amount of pro-IL-1β by 2.3±0.4-fold (figure 3C). In summary, neither commensal nor non-toxigenic *S. aureus* induced NF-κB activation or production of pro-IL-1β components of the TLR pathway.

### Effect of commensal, non-toxigenic and toxigenic bacteria on expression of NLRP3 in conjunctival goblet cells

**Figure 4:**
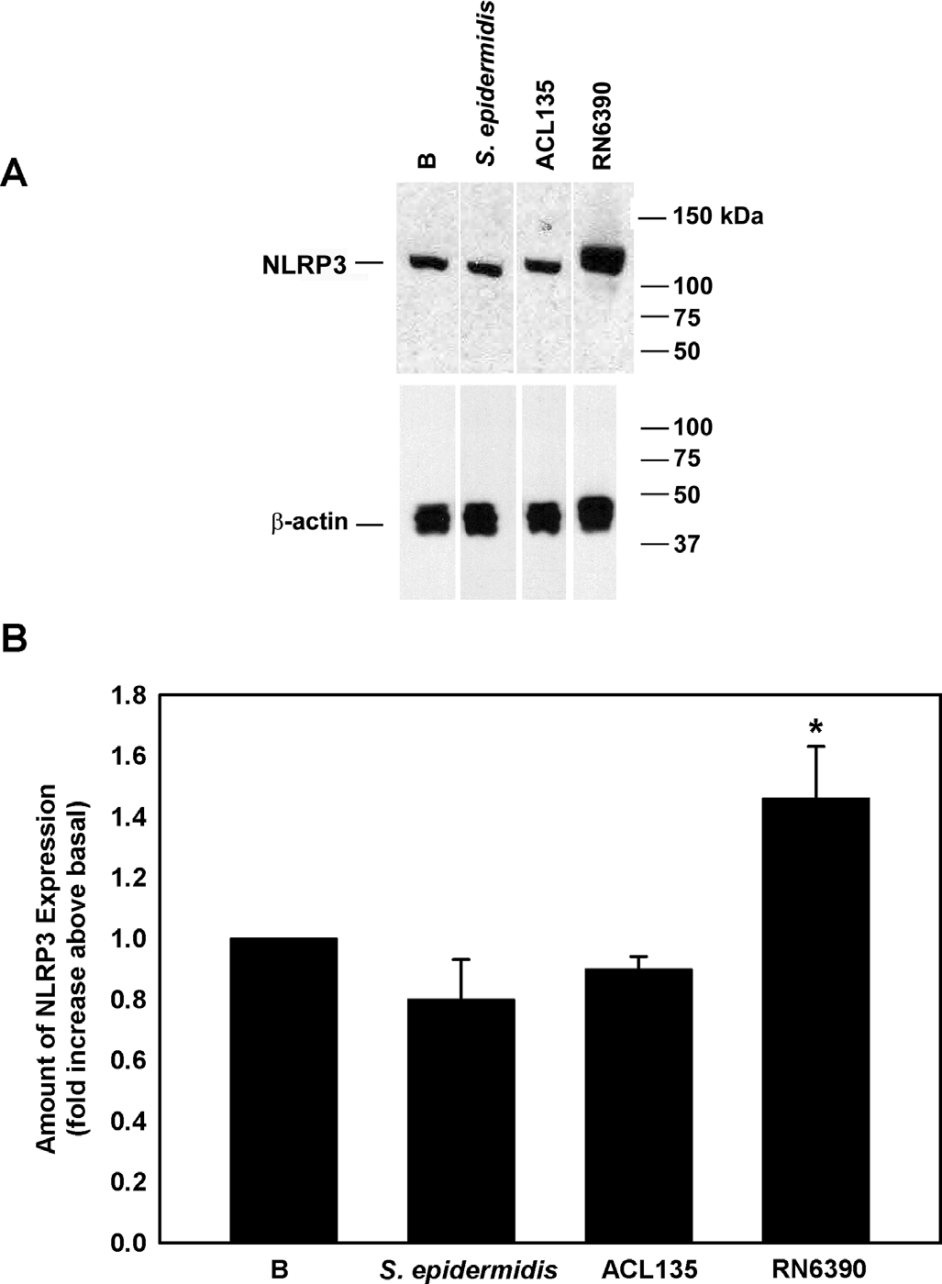
Effect of *Staphylococcus epidermidis*, *S. aureus* ACL 135 and *S. aureus* RN6390 on NLRP3 amount. Cultured human goblet cells were co-cultured for 4 hours with *S. epidermidis*, *S. aureus* ACL135 or *S. aureus* RN6390 at a multiplicity of infection of 20. The amount of NLRP3 was measured by western blot. A representative blot is shown in (A). The amount of NLRP3 was standardised to the amount of β-actin and data presented as fold increase above basal in (B**.**). Data are expressed as mean±SEM from three independent experiments. * indicates significance from basal (B). NLRP3, NOD-like receptor pyrin domain 3.

Western blot was used to assess NLRP3 expression in cell lysates prepared from human conjunctival goblet cells cultured (figure 4A,B) under the same conditions used to measure NF-κB activity (figure 3). When co-cultured with *S. epidermidis* or *S. aureus* ACL135 (figure 4A,B), there was no significant increase in NLRP3 expression detected in the goblet cells (0.8±0.1-fold and 0.9±0.04-fold over basal, respectively). A significant increase was only observed in goblet cells co-cultured with *S. aureus* RN6390 (1.5±0.2-fold over basal), the positive control. Exposure of goblet cells to commensal or non-toxigenic bacteria did not trigger increased expression of NLRP3, a component of the NLRP3 inflammasome.

### Toxigenic *S. aureus*-induced production of pro-IL-1β and release of mature IL-1β is NLRP3 dependent in conjunctival goblet cells

**Figure 5:**
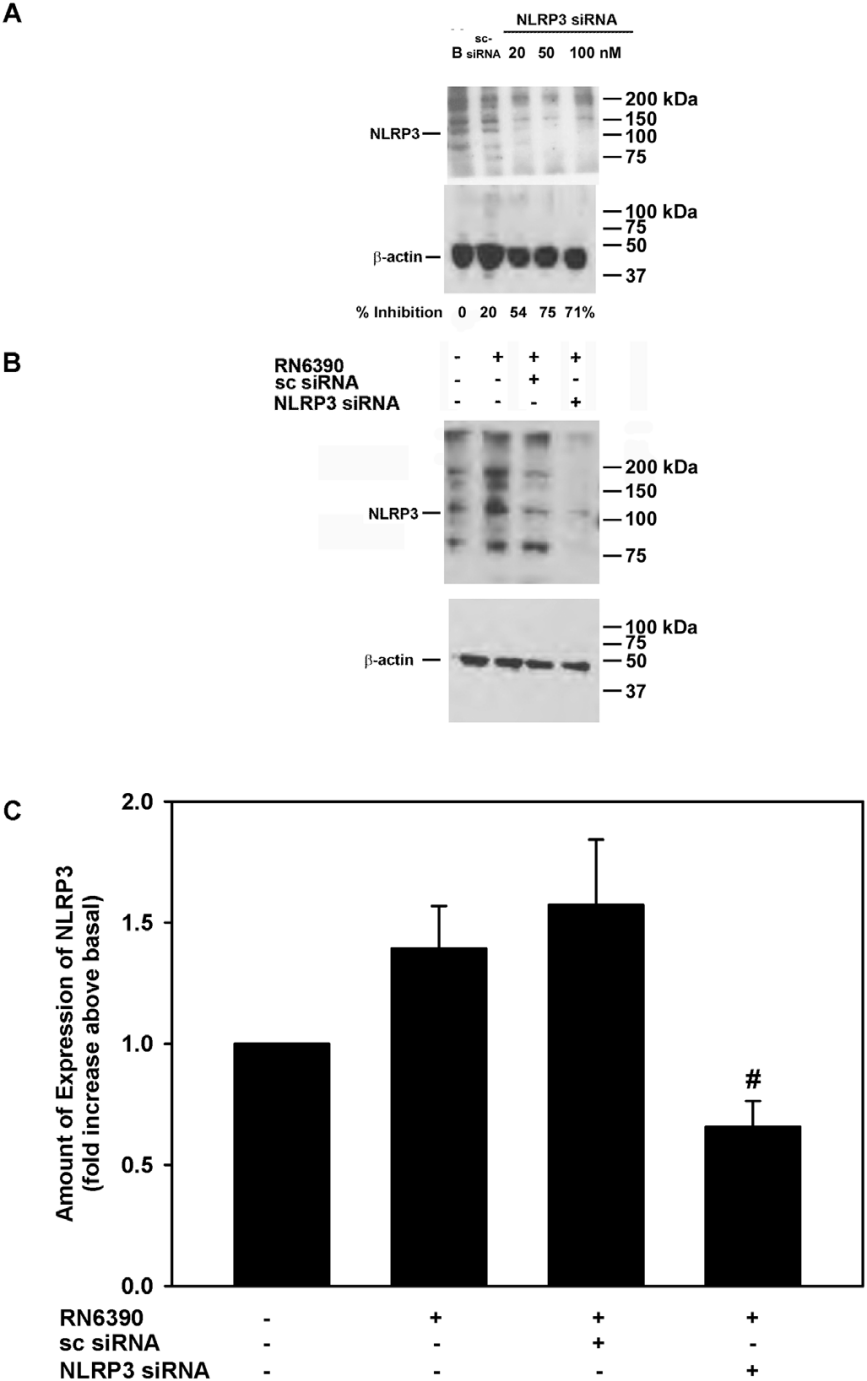
Effect of NLRP3 siRNA on *Staphylococcus aureus* RN6390-stimulated NLRP3 amount. Cultured human goblet cells were incubated for 48 hours with NLRP3 siRNA at increasing concentrations, and the amount of NLRP3 was determined by western blot analysis. A representative blot is shown in (A). Cultured human goblet cells were incubated with and without 50 nM NLRP3 siRNA or 100 nM sc-siRNA prior to co-culture for 4 hours with *S. aureus* RN6390 at a multiplicity of infection of 20. Representative blot is shown in (B). The amount of NLRP3 was standardised to the amount of β-actin and data presented as fold increase above basal in (C). Data are expressed as mean±SEM from three independent experiments. # indicates significance from incubation with RN6390 alone. NLRP3, NOD-like receptor pyrin domain 3; sc-siRNA, scrambled siRNA.

To determine whether the induction of pro-IL-1β and secretion of mature IL-1β by toxigenic *S. aureus* was dependent on NLRP3, we knocked down NLRP3 with a mixture of specific siRNAs. First-passage human conjunctival goblet cells were incubated for 72 hours with 20, 50 or 100 nM of NLRP3 siRNAs, or 100 nM of sc-siRNA, the negative control. Under basal conditions, NLRP3 protein was detected at the expected molecular weight of 117 kDa and incubation with sc-siRNA had minimal effects with only a 20% inhibition of NLRP3 protein levels (figure 5A). In contrast 20, 50 and 100 nM of NLRP3 siRNA decreased NLRP3 protein levels by 54%, 75% and 71%, respectively.

**Figure 6:**
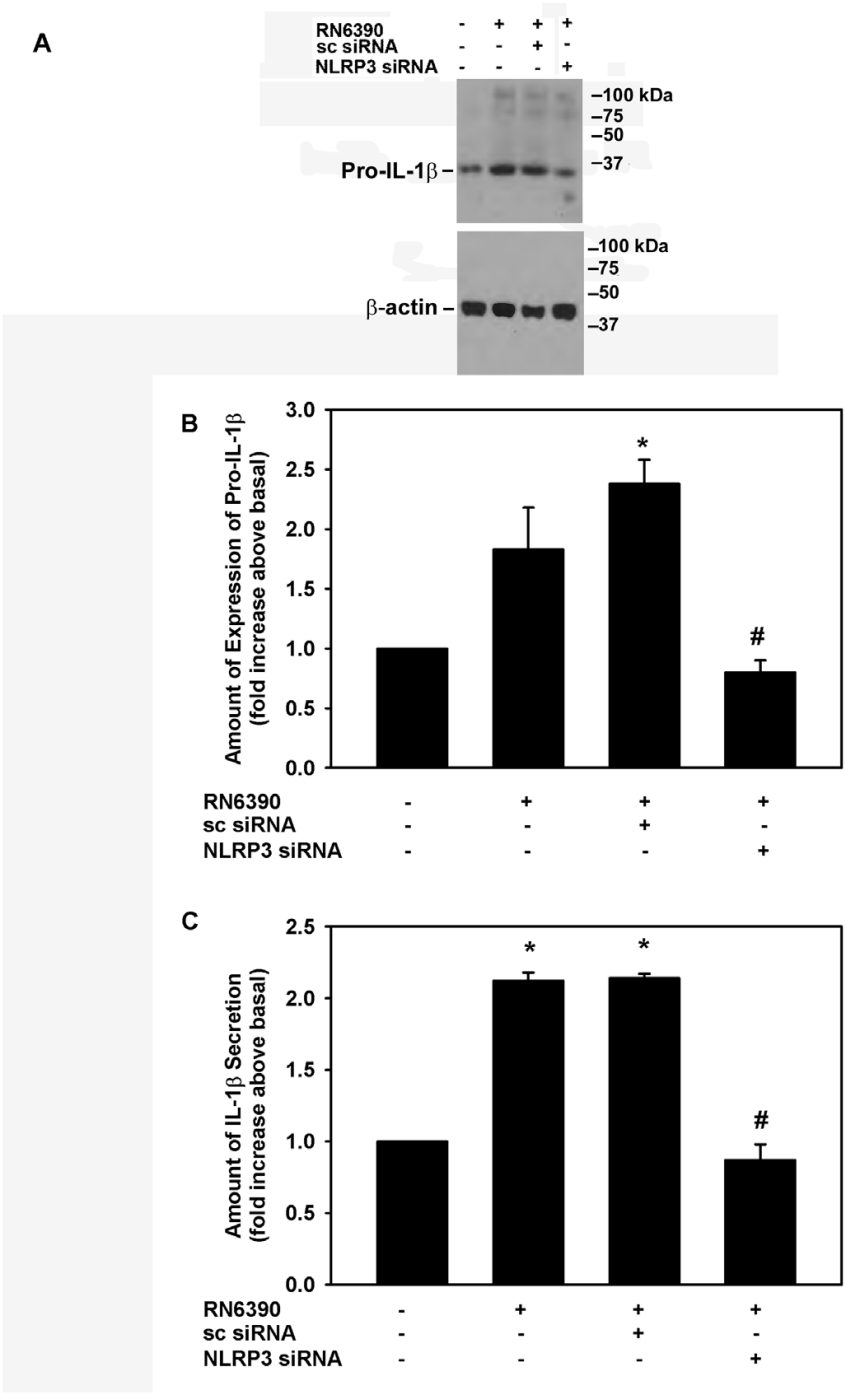
Effect of NLRP3 siRNA on *Staphylococcus aureus* RN6390-stimulated pro-IL-1β expression and mature IL-1β secretion. Cultured human goblet cells were incubated for 48 hours with 50 nM NLRP3 siRNA or 100 nM sc-siRNA prior to co-culture for 4 hours with *S. aureus* RN6390 at a multiplicity of infection of 20. The amount of pro-IL-1β was measured by western blot. A representative blot is shown in (A). The amount of pro-IL-1β was standardised to the amount of β-actin and data presented as fold increase over basal in (B). The amount of mature IL-1β secreted was determined by ELISA and is presented as fold increase over basal in (C). Data are expressed as mean±SEM from three independent experiments. * indicates significance from basal; # indicates significance from incubation with bacteria alone. IL, interleukin; NLRP3, NOD-like receptor pyrin domain 3; sc-siRNA, scrambled siRNA.

The addition of *S. aureus* RN6390 increased NLRP3 protein expression by 1.4±0.2-fold (Figure 5B and C). The use of sc-siRNA with cultured cells had no significant effect on S. *aureus*-induced NLRP3 expression. However, the use of 50 nM NLRP3 siRNAs in cultured goblet cells completely blocked the increase in NLRP3 protein expression and decreased it to below basal levels (figure 5B,C). In the same experiments, the expression of pro-IL-1β and secretion of mature IL-1β was determined (figure 6A-C). Under basal conditions, cultured goblet cells expressed a constitutive level of pro-IL-1β, but incubation with *S. aureus* RN6390 increased the amount of pro-IL-1β by 1.8±0.4-fold (figure 6A,B). *S. aureus* RN6390-induced expression of pro-IL-1β was completely abolished when NLRP3 was knocked down with siRNA (figure 6B).

Similarly to its effect on pro-IL-1β, NLRP3 knockdown also prevented the *S. aureus*-induced increase in the secretion of mature IL-1β. Under basal conditions, mature IL-1β was secreted and detected in the culture supernatants. Co-culture with *S. aureus* RN6390 increased this amount by 2.1±0.1-fold (figure 6C). Stimulation of mature IL-1 β secretion by *S. aureus* RN6390 was not altered by sc-siRNA, but was completely blocked by NLRP3 siRNAs. These data demonstrate in human conjunctival goblet cells that *S. aureus* RN6390-induced expression of pro-IL-1β and secretion of mature IL-1β is dependent on NLRP3 signalling.

### Effect of commensal, non-toxigenic and toxigenic bacteria on activation of caspase-1 and release of mature IL-1β in conjunctival goblet cells

**Figure 7:**
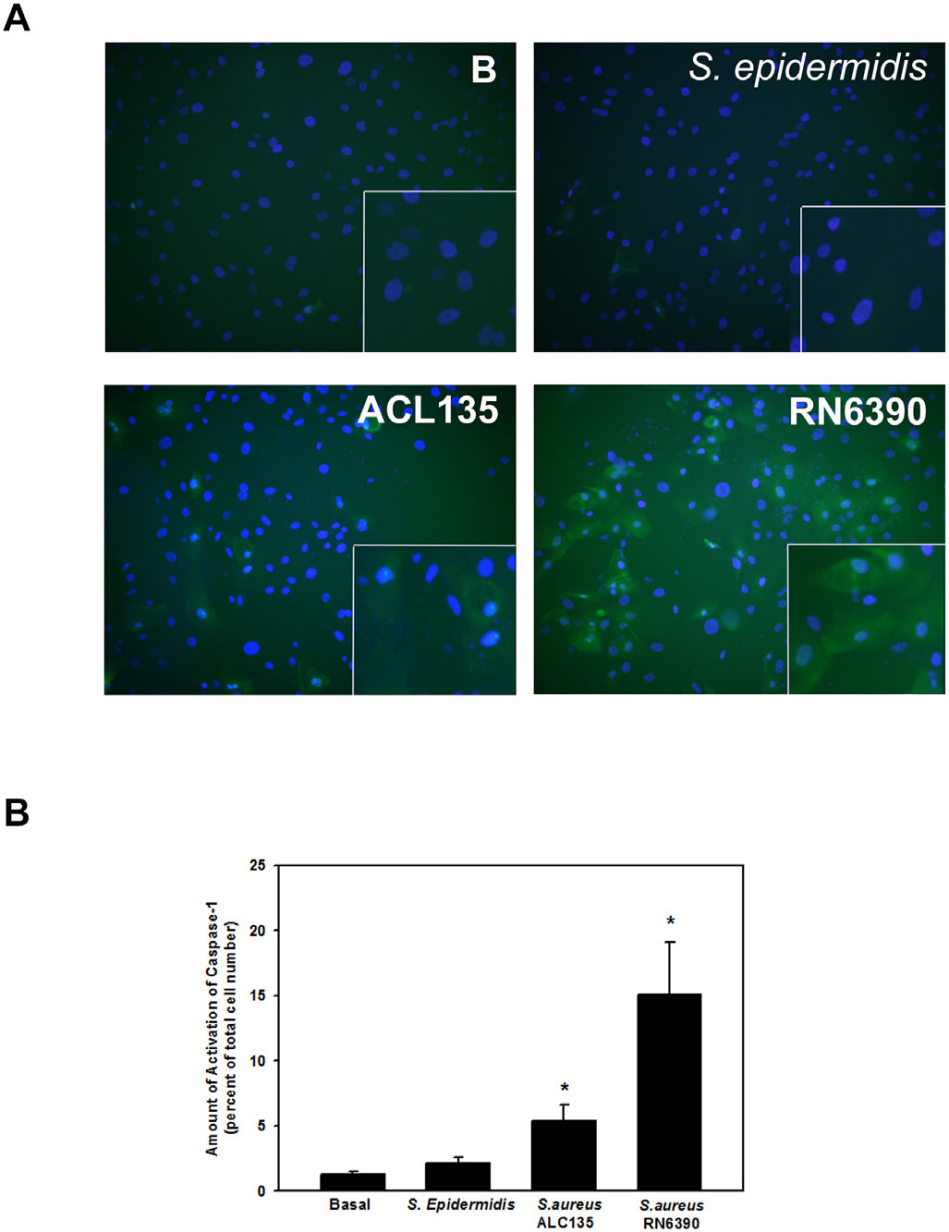
Effect of *Staphylococcus epidermidis*, *S. aureus* ACL 135 and *S. aureus* RN6390 on caspase-1 activity. Cultured human goblet cells were co-cultured for 4 hours with no addition (Basal, (B)), *S. epidermidis*, *S. aureus* ACL135 or *S. aureus* RN6390 at a multiplicity of infection of 20. The activity of caspase-1 was measured using a FLICA assay. Representative micrographs are shown in (A). Magnification ×400. Data shown in (B) are mean±SEM from three independent experiments. * indicates significance from basal (B).

Specific to *S. aureus* infections, the second step of NLRP3 inflammasome activation can be mediated by bacterial products such as peptidoglycans^36^ and/or bacterial toxins.^37^ As a measure of NLRP3 inflammasome activation, caspase-1 activity was evaluated using the FLICA probe FAM-YVAD-FMK that specifically labels active caspase-1,^9^ in first-passage human conjunctival goblet cells co-cultured with *S. epidermidis*, *S. aureus* ACL135 or *S. aureus* RN6390 at an MOI of 20 for 4 hours. There was no significant caspase-1 activity detected in goblet cells incubated with commensal *S. epidermidis* (figure 7A,B). Surprisingly, a fivefold increase in caspase-1 activity was observed in goblet cells co-cultured with non-toxigenic *S. aureus* ACL135 (5.4±1.2-fold over basal) (figure 7B). However, an even greater >15 fold increase in caspase-1 activity was observed in goblet cells co-cultured with the toxigenic *S. aureus* RN6390 (15.1±4.0-fold over basal).

**Figure 8:**
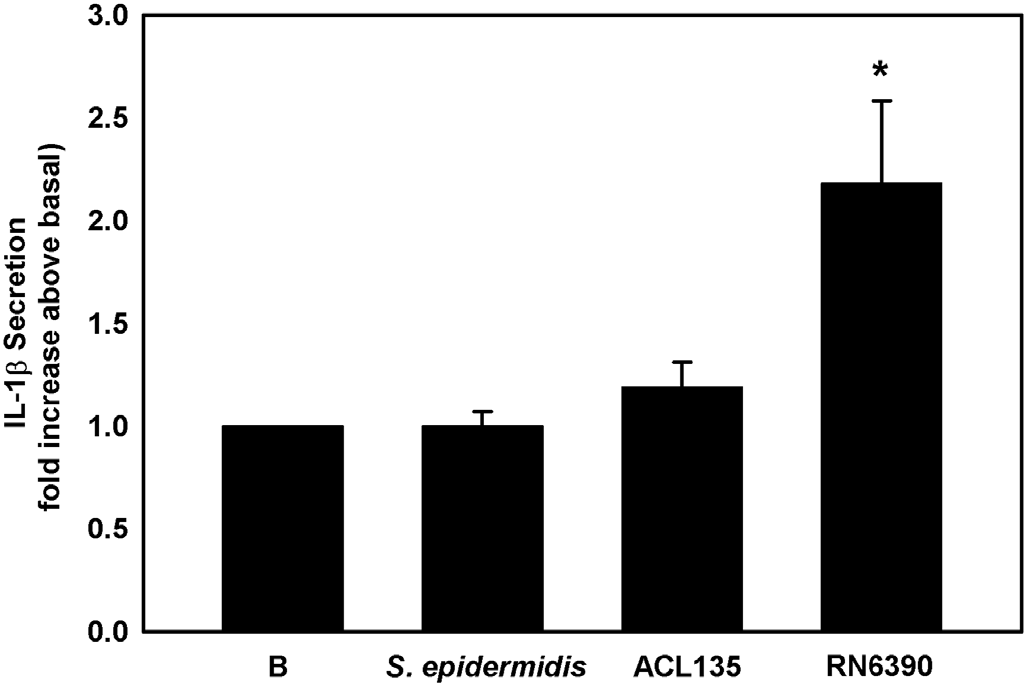
Effect of *Staphylococcus epidermidis*, *S. aureus* ACL 135 and *S. aureus* RN6390 on secretion of mature IL-1β. Cultured human goblet cells were co-cultured for 4 hours with *S. epidermidis*, *S. aureus* ACL135 or *S. aureus* RN6390 at a multiplicity of infection of 20. The amount of IL-1β secreted was measured by ELISA and presented as fold increase over basal. Data shown are mean±SEM from three independent experiments. * indicates significance from basal (B). IL, interleukin.

The final end product of NLRP3 inflammasome activation is the secretion of mature IL-1β. IL-1β secretion was measured by ELISA in supernatant of first-passage cultures of human conjunctival goblet cells co-cultured with *S. epidermidis*, *S. aureus* ACL135 or *S. aureus* RN6390 at an MOI of 20 for 4 hours (figure 8). There was no significant increase in IL-1β secretion from goblet cells co-cultured with the commensal *S. epidermidis* or the non-toxigenic *S. aureus* ACL135, when compared with basal levels (1.2±0.1-fold and 1.0±0.1-fold, respectively). However, the positive control toxigenic *S. aureus* RN6390 significantly increased the secretion of mature IL-1β (2.2±0.4-fold over basal). These data demonstrate that commensal and non-toxigenic bacteria cannot activate the NLRP3 inflammasome to stimulate production of mature IL-1β in human conjunctival goblet cells

### Effect of commensal, non-toxigenic and toxigenic bacteria on secretion of high-molecular-weight glycoconjugates by conjunctival goblet cells

**Figure 9:**
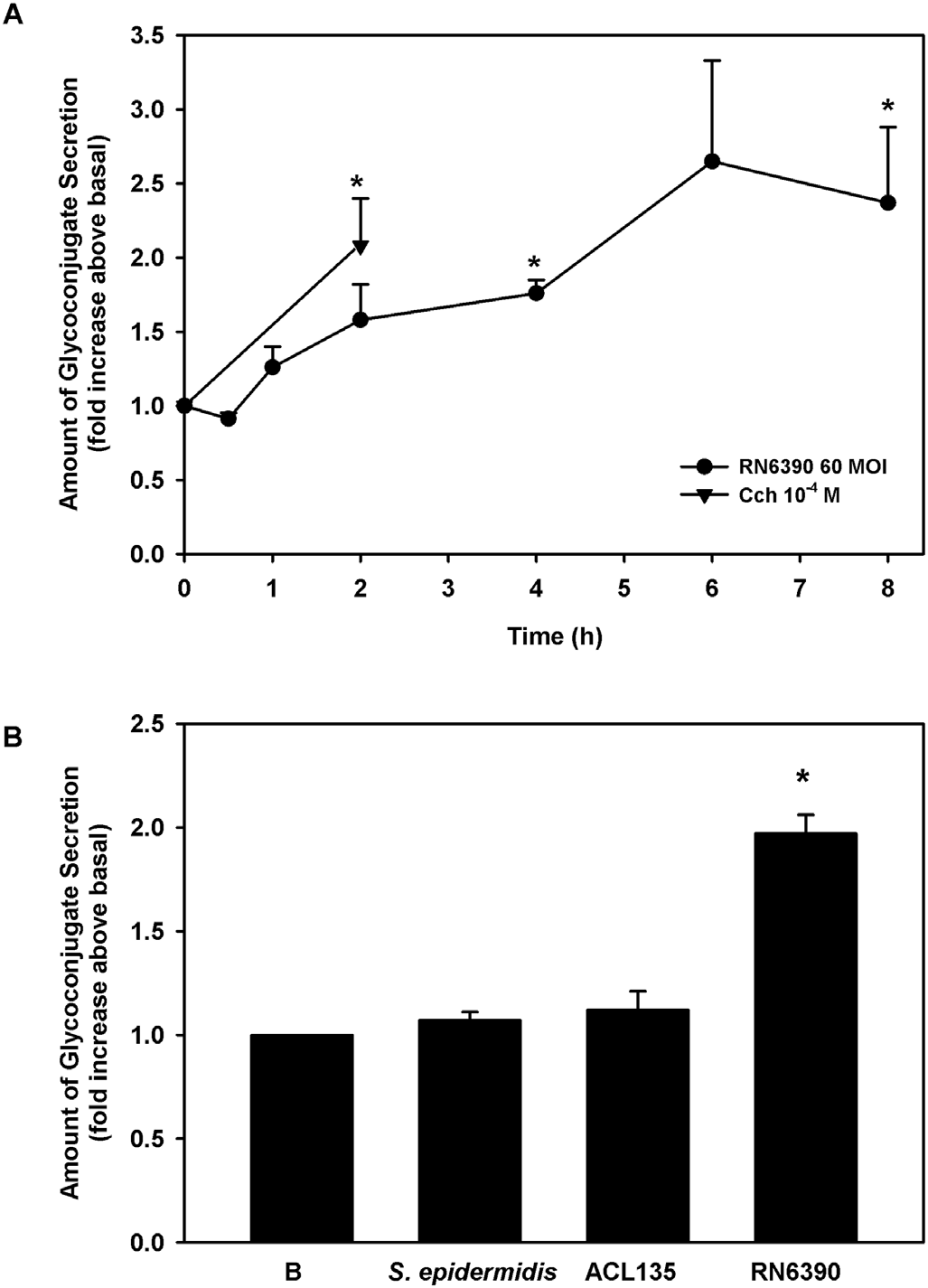
Effect of *Staphylococcus epidermidis*, *S. aureus* ACL 135 and *S. aureus* RN6390 on high-molecular-weight glycoconjugate secretion. (A) Human conjunctival goblet cells were co-cultured for 0–8 hours with *S. aureus* RN6390 at an MOI of 60 (circles) or incubated with the cholinergic agonist carbachol (Cch, triangles) at 10^−4^ M for 2 hours. Glycoconjugate secretion was measured by enzyme-linked lectin assay (ELLA) and presented as fold increase over basal. (B). Cultured human goblet cells were co-cultured for 4 hours with *S. epidermidis*, *S. aureus* ACL135 or *S. aureus* RN6390 at an MOI of 20. Glycoconjugate secretion was measured by ELLA and presented as fold increase above basal. Data shown are mean±SEM from three independent experiments. * indicates significance from basal (B). MOI, multiplicity of infection.

Human conjunctival goblet cells were co-cultured for 0–8 hours with *S. aureus* RN6390 at an MOI of 60. As a positive control, cultured cells were treated for 2 hours with a known stimulant of goblet cell secretion, the cholinergic agonist, carbachol at 10^−4^ M.^31 38 39^ The toxigenic *S. aureus* RN6390 induced conjunctival goblet cell glycoconjugate secretion in a time-dependent manner, beginning 1 hour after exposure, becoming significant after 4 hours (1.6±0.2-fold) and peaking at 2.4±0.5-fold by 8 hours (figure 9A).

Because significant levels of glycoconjugate secretion were detected at 4 hours and to minimise the impact of bacteria on cell viability, all subsequent secretion experiments were performed at 4 hours. To determine the ability of commensal *S. epidermidis* and non-toxigenic *S. aureus* ACL135 to stimulate goblet cell secretion, human conjunctival goblet cells were incubated with each strain at an MOI of 20 for 4 hours (figure 9B). Neither *S. aureus* ACL135 nor *S. epidermidis* induced goblet cell secretion above basal levels (1.1±0.1-fold and 1.1±0.04-fold, respectively), while the positive control, toxigenic *S. aureus* RN6390 significantly increased goblet cell secretion by 2.0±0.1-fold. Thus, neither commensal nor non-toxigenic bacteria can stimulate conjunctival goblet cell secretion.

## Discussion

Our results demonstrate that conjunctival goblet cells discriminate isogenic *S. aureus* strains based on expression of postexponential phase secreted products including toxins,^40^ as well as between pathogenic and commensal staphylococci. Of the strains tested, the commensal and non-toxigenic ones did not cause activation of the NLRP3 inflammasome and high-molecular-weight glycoprotein secretion. Only toxigenic *S. aureus,* the positive control, was effective. In most wet mucosal surfaces such as lung, intestine and colon, monocytes or macrophages mediate a response to toxigenic or commensal bacteria.^41–44^ However, it is possible that epithelial cells in these tissues are responsive to bacteria as well, but less is known about the inflammasome response of epithelial cells in the absence of immune effectors. In the skin, the keratinocytes respond to both commensal *S. epidermidis* 1457 and toxigenic *S. aureus* (113 ΔspA), by secretion of antimicrobial factors^45^; however, commensal bacteria induce a smaller response. While our data show no induction of the NLRP3 inflammasome or mucin secretion in conjunctival goblet cells exposed to commensal or non-toxogenic *S. aureus*, we cannot rule out that conjunctival goblet cells may respond to non-toxigenic and commensal bacteria when exposed to greater bacterial loads than tested here.

NLRP3 inflammasome activation is believed to be dependent on the concomitant activation of two pathways.^9^ The first pathway involves activation of an TLR receptor to increase synthesis of pro-IL-1β, NLRP3 and the other components of the NLRP3 inflammasome; the second pathway involves opening of an ion channel that triggers the activation of caspase-1. Activated caspase-1 then cleaves pro-IL-1β to produce mature IL-1β. In this study, we examined the components of both pathways, including NF-κB activation, production of pro-IL-1β and NLRP3 expression characteristic of the TLR pathway, and caspase-1 activation and release of mature IL-1β components of the NLRP3 pathway. In human conjunctival goblet cells, we found that neither commensal nor non-toxigenic strains triggered the activation of NF-κB, increased expression of pro-IL-1β and NLRP3, and secretion of mature IL-1β. To our surprise, though, exposure of human conjunctival goblet cells to non-toxigenic *S. aureus*, but not commensal *S. epidermidis*, resulted in significant activation of caspase-1. However, in the absence of NF-κB activation and production of pro-IL-1β, non-toxigenic bacteria were unable to stimulate the release of mature, active IL-1β. This result suggests that there are either alternative pathways for activation of caspase-1 or additional triggers that promote secretion of mature IL-1β. Known pathways include inhibition of GAPDH and glycolysis, activation of lipid metabolic pathways and stimulation of pyroptosis,^46^ and it will be of interest to determine their relevance to the observations made here.

Previous studies suggest that the ocular surface is normally maintained in a non-inflamed state, despite being in constant contact with bacteria from the skin and environment.^17^ The findings of this study demonstrating that neither commensal nor non-toxigenic bacteria trigger NLRP3 inflammasome activation or mucin secretion in conjunctival goblet cells, are in agreement with this concept. Secretion of mature IL-1β and mucin from conjunctival goblet cells was induced only by the toxigenic strain of *S. aureus*, similar to those frequently causing infection

In addition to conjunctiva, goblet cells are present in airways, the gastrointestinal tract and within the nasal epithelium. These cells are packed with secretory granules filled with large gel-forming mucins, MUC5AC, MUC5B or MUC2, which protect the epithelium by entrapping bacteria and preventing bacterial contact with the epithelium.^40 47–49^ Goblet cells also secrete key proteins involved in innate defence, with secreted proteins from gastrointestinal goblet cells being the most thoroughly studied.^50^ Proteins secreted include Ca^2+^ channel protein CLCA1; Fc globulin-binding protein (FCGBP), which binds and crosslinks mucus proteins; ZG16, which binds Gram-positive bacteria; AGR2, an ER residential protein; and trefoil factor 3 (TFF3). These proteins are secreted in high concentration into the mucus. We show here that toxigenic bacteria activate protective conjunctival goblet cell mucous secretion, in addition to activating the NLRP3 inflammasome. Thus, goblet cells have two different protective mechanisms against toxigenic bacteria that threaten the ocular surface. Neither of these protective mechanisms were activated by the commensal or non-toxigenic bacteria strains used.

In summary, we conclude that (1) conjunctival goblet cells do not respond to the presence of commensal and non-toxigenic bacteria to activate two protective functions of the goblet cells activation of the NLRP3 inflammasome and stimulation of goblet cell mucous secretion and (2) in contrast NLRP3 plays a critical role as a sensor of pathogenic bacteria in goblet cells of the conjunctiva epithelium, and that activation of the NLRP3 inflammasome mediates secretion of both mature IL-1β and large secretory mucins from human conjunctival goblet cells.
